# Nicotine Regulates LPS‐Induced Inflammatory Responses in HMC3 Microglia and Exerts Neuronal Protection

**DOI:** 10.1155/mi/4652344

**Published:** 2026-01-21

**Authors:** Yuhan Qin, Xiaohui Yan, Yanbo Luo, Hongjuan Wang, Yushan Tian, Xiaqing Wu, Huan Chen, Hongwei Hou, Qingyuan Hu

**Affiliations:** ^1^ Beijing Life Science Academy, Beijing, 102209, China; ^2^ Key Laboratory of Tobacco Biological Effects, China National Tobacco Quality Supervision and Test Center, No. 6 Cuizhu Street, New & High-tech Industry Development Zone, Zhengzhou, 450001, China; ^3^ Department of Chemistry, Key Laboratory of Bioorganic Phosphorus Chemistry and Chemical Biology, Tsinghua University, Beijing, 100084, China, tsinghua.edu.cn

**Keywords:** α7 nAChR, cell coculture, HMC3, lipopolysaccharide, microglia, neurodegenerative disease, neuroinflammation, neuron, nicotine, PI3K

## Abstract

Microglia‐mediated neuroimmune responses have been implicated in central nervous system injury and disease pathogenesis. The α7 nicotinic acetylcholine receptor (α7 nAChR), which is expressed on microglia and participates in microenvironment interactions, is a key mediator of the cholinergic anti‐inflammatory pathway. Nicotine activates the α7 nAChR, which may mediate the inflammation of microglia. This study aims to explore the modulatory effects of nicotine on neuroinflammation and its potential indirect neuroprotective effects using an in vitro microglial cell inflammation model. In our study, inflammatory phenotype indicators and molecular mechanisms of HMC3 cells were analyzed. Furthermore, an HMC3 microglia‐SH‐SY5Y neuronal coculture system was constructed to investigate the indirect neuroprotective effects of nicotine. The results demonstrated that nicotine exerted an inhibitory effect on the lipopolysaccharide‐induced HMC3 microglia inflammation, promoted the release of neurotrophic factors, and neuronal survival by altering the immune environment. These effects appear to be mediated through the activation of α7 nAChR, leading to an increase in phosphorylation of PI3K. This study provides important insights into the immunomodulatory functions of low‐concentration nicotine in the nervous system and contributes to a deeper understanding of its potential therapeutic applications.

## 1. Introduction

Neurodegenerative diseases, such as Alzheimer’s and Parkinson’s, are characterized by the loss and dysfunction of neurons in the central nervous system, leading to cognitive and motor impairment. Activation of innate immunity, including microglia‐mediated responses, contributes to these conditions, triggering neurotoxic pathways and progressive degeneration [1, 2]. Modulating the immune system could be a promising approach for mitigating such conditions. Nicotine, a unique alkaloid found in tobacco, has been found to have beneficial physiological effects, including immune regulation and neuroprotection [3–7]. Epidemiological studies suggest that smoking behavior is associated with a lower risk of Parkinson’s disease [8, 9], possibly due to nicotine’s anti‐inflammatory effects, which can also protect neurons from glutamate excitotoxicity [10, 11]. However, current research on nicotine’s ability to regulate the central nervous system’s immune inflammation is limited and inconclusive.

To investigate the potential neuroinflammatory inhibitory and indirect neuroprotective effects of nicotine, we established a coculture system consisting of human‐derived microglia and neurons using the HMC3 human microglial cell line as a model. Microglia are the immune cells that reside in the central nervous system and interact with the microenvironment through various receptors, including the α7 nicotinic acetylcholine receptor (α7 nAChR), which is a crucial target of the cholinergic anti‐inflammatory pathway [12–16]. In addition, PI3K, an intracellular phosphatidylinositol kinase that participates in cell growth, proliferation, apoptosis, and autophagy, also contributes to neuro‐immune regulation [6, 17, 18].

In the current study, we developed an in vitro model of neuroinflammation using lipopolysaccharide (LPS)‐induced HMC3 cells. We investigated the effects of nicotine intervention on the physiological activity and inflammatory level of HMC3 cells by measuring various indicators, including inflammatory factors, brain‐derived neurotrophic factor (BDNF), glia‐derived neurotrophic factor (GDNF), and nitric oxide (NO). Furthermore, we assessed the transcription levels of α7 nAChR in the cells and the phosphorylation levels of PI3K protein to gain further insight into the possible regulatory mechanisms of nicotine in the inflammatory response of HMC3 cells.

The coculture system used in this study provides an ideal model for investigating the effects of microglia‐neuron interactions. By utilizing transwell chambers, we were able to separate microglia and neurons while still enabling the exchange of signals and factors. This coculture system enabled us to investigate the effects of nicotine on both microglia and neurons and explore its potential indirect neuroprotective effects by modulating the inflammatory response of microglia. The system provides a more accurate representation of the in vivo microenvironment and allows for a more comprehensive understanding of the effects of nicotine on the neuroinflammatory response.

In conclusion, neurodegenerative diseases pose a significant challenge to public health, and modulating the immune system offers a promising approach to mitigating these conditions. Nicotine has been shown to have significant anti‐inflammatory effects and can protect neurons from glutamate excitotoxicity. However, research on its role in regulating central nervous system immune inflammation is limited and inconclusive. This study aimed to investigate nicotine’s potential neuroinflammatory inhibitory and indirect neuroprotective effects. The results could enhance our understanding of the mechanisms underlying nicotine’s effects on the central nervous system and provide insights into novel therapeutic strategies for neurodegenerative diseases.

## 2. Materials and Methods

### 2.1. Materials

Nicotine (>99% purity) was obtained from the National Tobacco Quality Supervision and Test Center (Zhengzhou, China). LPS was acquired from Sigma–Aldrich (USA). GTS‐21 dihydrochloride (an α7 nAChR‐specific agonist) and methyllycaconitine citrate (MLA) (an α7 nAChR‐specific inhibitor) were purchased from MedChemExpress (NJ, USA).

### 2.2. Cell Culture and Treatment

The HMC3 cell line was purchased from Procell Life Science & Technology (Wuhan, China) and cultured in Minimum Essential Medium (MEM) (Procell, Wuhan, China) supplemented with 10% fetal bovine serum (FBS) (Gibco, CA, USA). The SH‐SY5Y human neuroblastoma cell line was obtained from the Key Laboratory of Tobacco Biological Effects (Zhengzhou, China) and grown in a medium prepared through mixing of half MEM and half Ham’s F‐12K (Kaighn’s) Medium (Gibco, CA, USA) supplemented with 10% FBS and 10 µM sodium pyruvate. Cells were cultured at 37°C and 5% CO_2_ in a humidified incubator. The medium was replaced every 2 days. HMC3 cells were exposed to different concentrations of LPS (0, 0.1, 1, 10, and 100 μg/mL) for 24 h. In subsequent experiments, HMC3 cells were exposed to 10 μg/mL LPS for 24 h.

Prior to administration, HMC3 cells (2 × 10^5^/mL) were added to well plates. After 12 h of starvation treatment, cultures were aspirated from the well plates for subsequent experimentation. The cells were separated into seven treatment groups: control group (Con), model group (Mod), nicotine administration groups (low, medium, and high doses) (Low, Med, High), positive control group (Pc), and negative control group (Nc). The cells in the blank control group were incubated with serum‐free MEM; the model control group was incubated with MEM medium together with LPS at a concentration of 10 μg/mL. The nicotine administration groups and positive control group were incubated with MEM medium supplemented with concentrations of 1, 10, and 100 μM nicotine and 100 μM GTS‐21 for 1 h, respectively, followed by the addition of MEM medium containing 10 μg/mL LPS to further the incubation. The negative control group was incubated with 10 μM MLA for 30 min, followed by 100 μM nicotine for 1 h, and finally followed by the addition of 10 μg/mL LPS to the MEM medium. All seven groups had their culture medium, and cells were harvested after 24 h of incubation. The experimental groups and design are shown in Figure 1a.

Figure 1Experimental grouping and pretreatment. (a) The experiment separated cells into seven groups: blank control group (Con), model control group (Mod), low nicotine experimental group (Low), medium nicotine experimental group (Med), high nicotine experimental group (High), positive control group (Pc), and negative control group (Nc). The dosing arrangements were made according to different dosing methods. (b) Detection of the effects of LPS (0.01, 0.1, 1, 10, and 100 μg/mL) on the viability of HMC3 cells. (c) Detection of the effects of nicotine (1, 10, 100, 1000, and 2000 μM) on the viability of HMC3 cells. p(Con vs. 2000) = 0.0004. (d) HMC3 cells were induced with different concentrations of LPS (0.1, 1, 10, and 100 μg/mL) for 24 h, and the IL‐6 content in the cell supernatant was measured. p(Con vs. 1) < 0.0001, p(Con vs. 10) = 0.0057, p(Con vs. 100) < 0.0001. (e) Cells were pretreated with different concentrations of nicotine (1, 10, and 100 μM) solution alongside 100 μM GTS‐21 solution for 1 h, respectively, and induced with LPS for 24 h. The content of IL‐6 in their cell supernatants was measured. p(Mod vs. Con) <0.0001, p(Mod vs. Low) = 0.0001, p(Mod vs. Med) = 0.0067, p(Mod vs. High) = 0.0055, p(Mod vs. Pc) = 0.0010.

**Figure:**
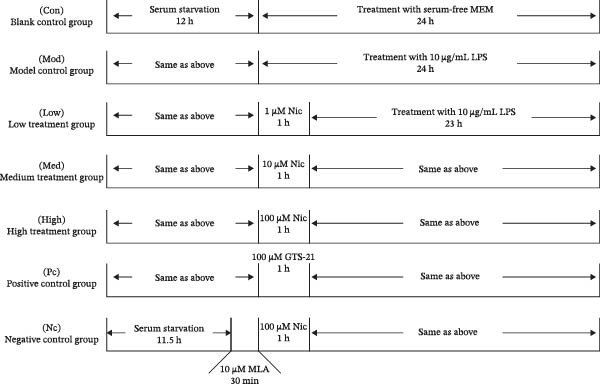
(a)

**Figure:**
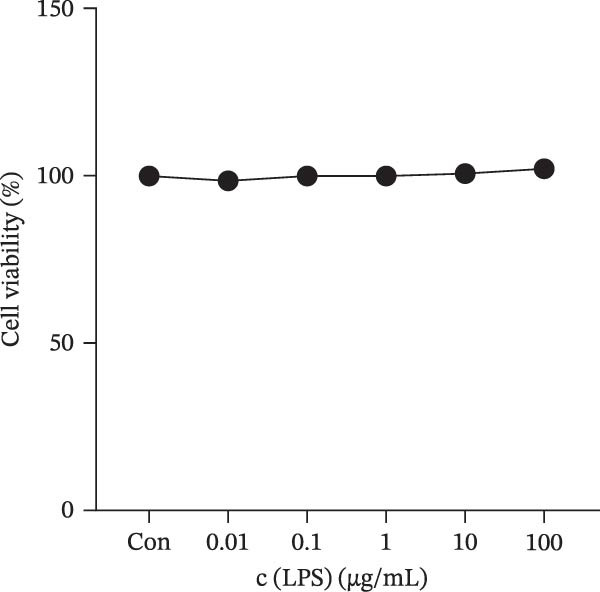
(b)

**Figure:**
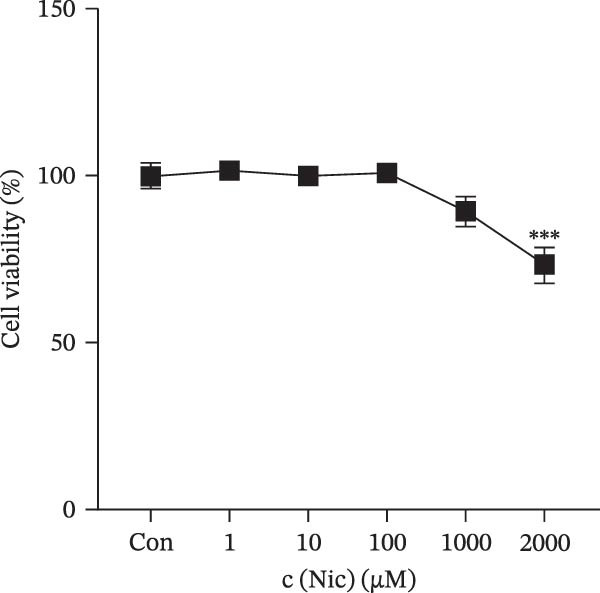
(c)

**Figure:**
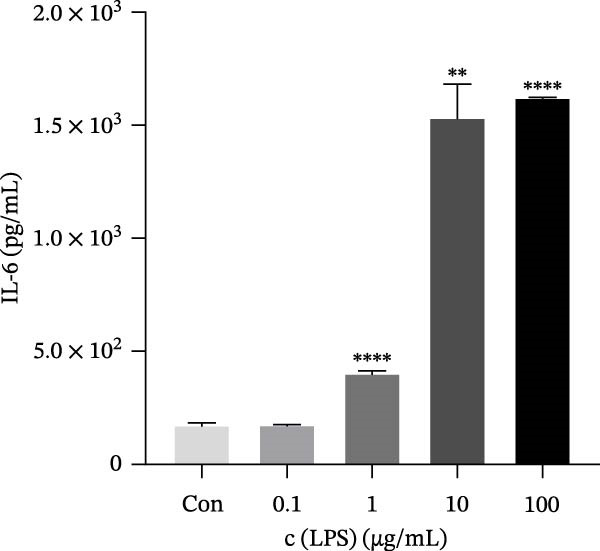
(d)

**Figure:**
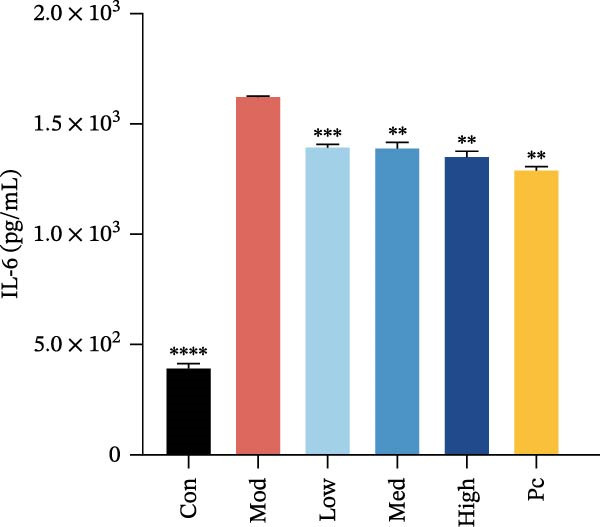
(e)

### 2.3. Cell Viability Assay

The Cell Counting Kit‐8 was used to assess HMC3 cell viability. After cells were inoculated into a 96‐well plate and cultured with different drugs according to our previous design. After treatment, 10% CCK‐8 solution (DOJINDO, Shanghai, China) was added for 3.5 h. An MTT assay was used to evaluate the viability of the SH‐SY5Y cells. Cells were incubated with 20% MTT solution (Beyotime, Shanghai, China) for 3.5 h. DMSO was then used to dissolve crystals to halt the reaction. Finally, the absorbance of the cells at 450 nm was measured using a multimode reader (TECAN, CH).

### 2.4. Enzyme‐Linked Immunosorbent Assay

After treatment, the HMC3 cells were collected and centrifuged to obtain the supernatant, then the supernatant was used to detect the content of IL‐6 (FineTest, Wuhan, China), BDNF (Abcam, Shanghai, China), and GNDF (Abcam, Shanghai, China) according to the manufacturer’s instructions.

### 2.5. Cytokine and Chemokine Assay

The levels of 27 human cytokines and chemokines in the HMC3 cell‐culture supernatant were determined using the Bio‐Plex Pro Human Cytokine 27‐plex Assay (BIO‐RAD, CA, USA), according to the manufacturer’s instructions. We used the Bio‐Plex 200 system for measurement.

The 27 cytokines and chemokines assessed include basic fibroblast growth factor (FGF), eotaxin, granulocyte colony‐stimulating factor (G‐CSF), granulocyte‐macrophage CSF (GM‐CSF), interferon‐gamma (IFN‐γ), interleukin‐1β (IL‐1β), IL‐1α, IL‐2, IL‐4, IL‐5, IL‐6, IL‐7, IL‐8, IL‐9, IL‐10, IL‐12(p70), IL‐13, IL‐15, IL‐17, γ‐interferon‐inducible protein‐10 (IP‐10), monocyte chemoattractant protein‐1 (MCP‐1), macrophage inflammatory protein‐1α (MIP‐1α), MIP‐1β, platelet‐derived growth factor‐BB (PDGF‐BB), regulated upon activation normal T cell expressed and secreted (RANTES), tumor necrosis factor (TNF)‐α, and vascular endothelial growth factor (VEGF).

### 2.6. Griess Reagent Kit for Nitrite Determination

NO is a molecular mediator of many physiological processes. Detection of nitrite formed by spontaneous oxidation of NO under physiological conditions can be accomplished via spectrophotometry. According to the manufacturer’s instructions, we used the Griess kit (Thermo Fisher, MA, USA) to detect nitrite in the cell supernatant.

### 2.7. NO Synthase Activity Assay

A kit (Beyotime, Shanghai, China) provides a suitable method for detecting the activity of NO synthase in living cells by fluorescence under physiological conditions. The kit utilizes a NO fluorescence detection probe, DAF‐FM DA (3‐amino, 4‐aminomethyl‐2′7′‐difluorescein, diacetate), which can penetrate the cell membrane in order to detect the amount of NO that can be formed through catalysis by intracellular NO synthase under conditions with sufficient substrate, illustrating the activity of NO synthase.

### 2.8. mRNA Extraction and qPCR

According to the manufacturer’s instructions, extraction of total RNA from microglia‐enriched cultures was accomplished using a SteadyPure Universal RNA Extraction Kit (Accurate, Hunan, China). The samples were dissolved in RNase‐free water, and the RNA content was quantified using a NanoDrop ND‐2000 spectrophotometer (NanoDrop Technologies, Wilmington, DE). The extracted RNA was reverse transcribed into single‐stranded cDNA using Evo M‐MLVRT Premix (Accurate, Hunan, China) according to the manufacturer’s directions using Evo M‐MLVRTase, dNTPs, Oligo (dT)18 Primer, Random 6 mers Primer, and RNase inhibitor. Reverse transcription reactions were performed using a Gene Amplification Apparatus (Eastwin, Beijing, China) at 37°C for 15 min and 85°C for 5 s.

qPCR reactions were performed in a total volume of 20 μL, containing 2 μL of sample cDNA, 2 × SYBR Green Pro TaqHS Premix (Accurate, Hunan, China), and 0.2 μM each of the forward and reverse primers (Accurate, Hunan, China) (Table 1). qPCR cycling was performed on a Light Cycler 96 (Roche, Indianapolis, IN) with a 30‐min preincubation at 95°C followed by 40 cycles of 5 s at 95°C, and 30 s at 60°C. The validity of the primer was confirmed using serially diluted cDNA, which established a standard curve. To determine mRNA expression levels, the comparative CT method was utilized [19]. The amount of target mRNA in each sample was then normalized to the reference gene GAPDH.

**Table 1:** List of primers used for qPCR.

Target gene	Forward primer sequence (5′–3′)	Reverse primer sequence (5′–3′)	Accession no.
α7 nAChR	GGACCAGCCTCCGTAA	ACTCGCAACCACTCACC	NM_000746
GAPDH	GCACCGTCAAGGCTGAGAAC	TGGTGAAGACGCCAGTGGA	NM_002046

### 2.9. Protein Extraction and Western Blot

Each group of HMC3 cells was collected 4 h after LPS administration, and the total intracellular protein was collected by centrifugation at 12,000 rpm for 20 min at 4°C using RIPA cell lysate (Beyotime Biotechnology, Shanghai, China). The protein concentration of each group was determined and equalized using a BCA kit (Solarbio, Beijing, China). The protein samples from each group were mixed with 4× loading buffer (Beyotime Biotechnology, Shanghai, China) and heated to 100°C for 10 min.

Next, the total protein was separated through sodium dodecyl sulfate polyacrylamide gel electrophoresis (Bio‐Rad, Hercules, CA, USA) and transferred to a polyvinylidenedifluoride membrane (Sigma–Aldrich, USA). The membranes were then blocked using 5% (w/v) skimmed milk in 1 × TBST (Tris‐buffered saline‐0.1% Tween) (Solarbio, Beijing, China) for 2 h at room temperature. The membranes were subsequently incubated with the following primary antibodies: Phospho‐PI3 Kinase p85 /p55 Rabbit mAb (E3U1H) (1:1000, #17366, Cell Signaling Technology, UK), PI3 Kinase p110α Rabbit mAb (C73F8) (1:1000, #4249, Cell Signaling Technology, UK), and GAPDH (1:10,000, Abcam, Shanghai, China), respectively, overnight at 4°C. The membranes were then incubated with horseradish peroxidase (HRP)‐conjugated goat anti‐rabbit lgG (1:3000, #7074, Cell Signaling Technology, UK) for 1 h at room temperature. Finally, protein bands were developed using an ECL kit (TaKaRa, Beijing, China), and photographs were quantified using the VosionCapt system.

### 2.10. Construction of the Coculture System

We used transwell (Corning, NYC, USA) plates to construct a microglia‐neuron coculture system. Human neuroblastoma cells SH‐SY5Y were placed into transwell chambers, and HMC3 cells were added into culture plates (Figure 2e). After separate overnight incubations, transwell chambers were placed into the culture plates and separated into groups for drug administration. After 24 h, the transwell chambers were removed, and the culture fluid was aspirated from the chambers. The MTT method was then used to assay the fluid and SH‐SY5Y neuronal cell activity in the chambers.

Figure 2Detection of molecular regulatory indicators from HMC3 cells and construction of a coculture model. (a) Intracellular α7 nAChR transcript levels were detected via qPCR. p(Mod vs. Low) = 0.0036, p(Mod vs. Med) = 0.0099, p(Mod vs. High) = 0.0370, p(Mod vs. Pc) = 0.0137. (b) The expression levels of intracellular PI3K and its phosphorylated product, p‐PI3K, were detected by WB. (c) The ratio of p‐PI3K to PI3K in HMC3 cells. p(LPS vs. Nic+LPS) = 0.0228. (d) Within the coculture system, the activity of each group of SH‐SY5Y cells was examined using the MTT method. p(LPS vs. Con) = < 0.0001, p(LPS vs. Nic+LPS) = 0.0003. (e) To construct a human‐derived neural cell coculture system, transwell chambers with a pore size of 0.4 μm were utilized. SH‐SY5Y cells were inoculated into the upper chamber, and HMC3 cells were inoculated into the lower chamber.

**Figure:**
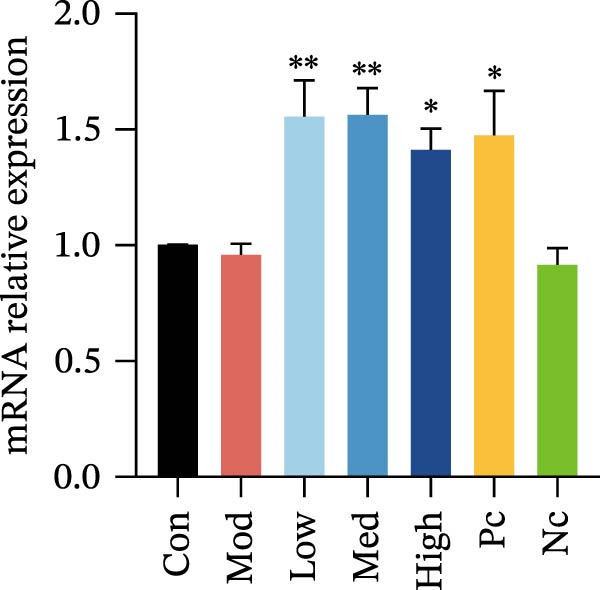
(a)

**Figure:**
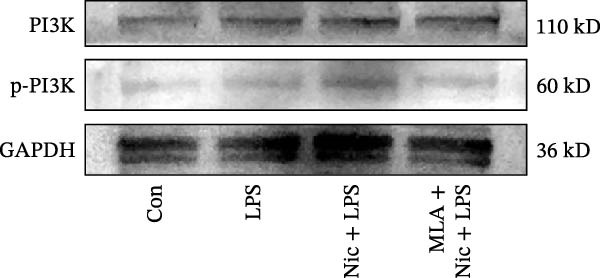
(b)

**Figure:**
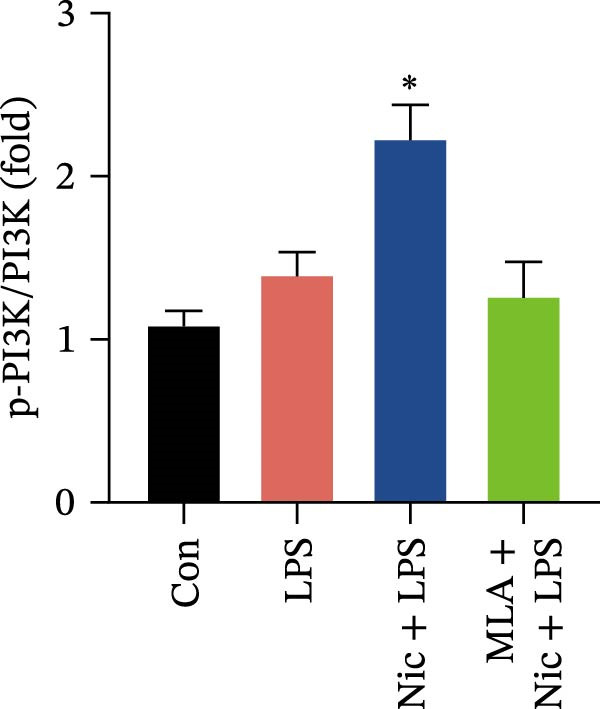
(c)

**Figure:**
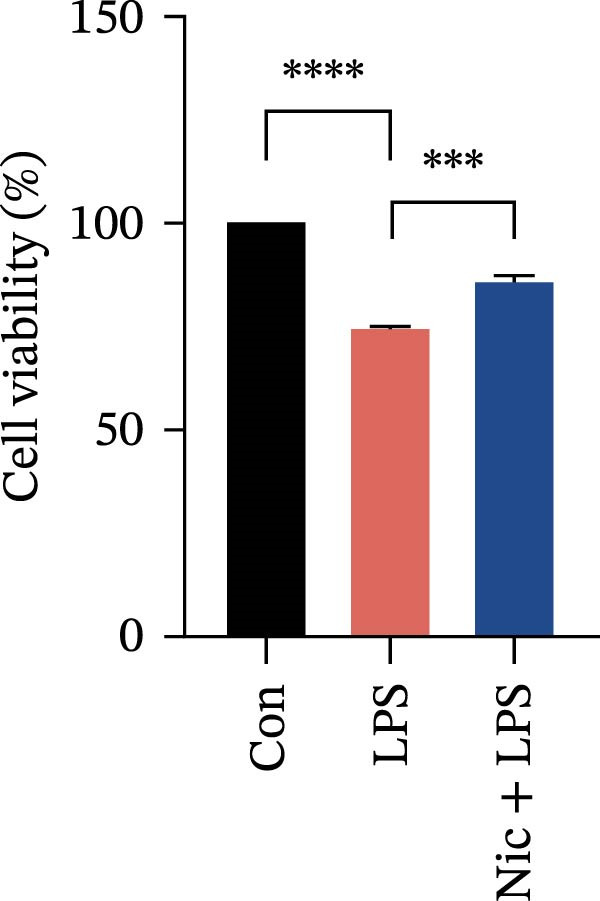
(d)

**Figure:**
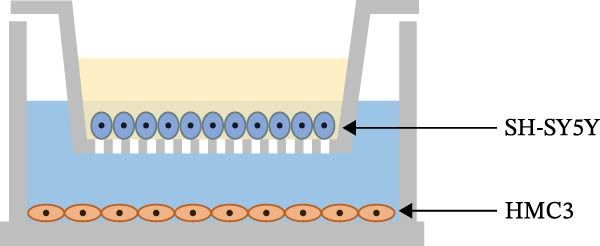
(e)

### 2.11. Statistical Analysis

Statistical analysis was performed using Software Prism 8 (GraphPad Software). The significance of differences between groups was determined through one‐way analysis of variance (one‐way ANOVA). Results were presented as mean ±standard deviation (SD). All experiments were performed at least three times, and statistical significance was defined as *p* < 0.05.  ^∗^ *p* < 0.05,  ^∗∗^ *p* < 0.01,  ^∗∗∗^ *p* < 0.001,  ^∗∗∗∗^ *p* < 0.001.

## 3. Results

### 3.1. Effect of LPS and Nicotine on HMC3 Cell Viability

The CCK‐8 method was used to explore the potential toxicity of LPS and nicotine on the HMC3 microglia. The results are shown in Figure 1, which indicates that LPS at concentrations ranging from 0.01 to 100 μg/mL and nicotine at 1–1000 μM do not impact HMC3 cell viability at the corresponding treatment durations. After treatment with nicotine (2000 μM), the activity of HMC3 cells was significantly decreased (Figure 1b,c), which indicating that high concentrations of nicotine are cytotoxic to HMC3 cells and the concentrations of nicotine should be controlled within the range not exceeding 1000 μM for the following experiments.

### 3.2. Nicotine Improved LPS‐Induced Inflammation in HMC3 Cells

To observe the inflammatory model in vitro, LPS was employed to coculture with HMC3 cells in 24‐well plates for 24 h. The results demonstrated that LPS at concentrations of 1, 10, and 100 μg/mL promoted production of IL‐6 from the cells compared to the blank control group (*p* < 0.05). Furthermore, the contents of IL‐6 were increased dramatically after coculture with LPS at concentrations of 10 and 100 μg/mL, and its effects were similar. Therefore, the following experiments employed LPS at 10 μg/mL in our HMC3 cell inflammation model (Figure 1d).

After HMC3 cells were pretreated with various concentrations (1, 10, 100 μM) of nicotine and 100 μM GTS‐21 (positive control group) for 1 h, the cells were then treated with LPS for 24 h. The supernatant was detected, and the results demonstrated that after LPS induction, the release of IL‐6 was obviously increased (*p* < 0.05) and nicotine pretreatment reduced the release of cellular IL‐6 compared to the model control group (*p* < 0.05). The effect of nicotine was similar to that of the positive drug GTS‐21 (Figure 1e), indicating the anti‐inflammation of nicotine. Therefore, 1, 10, and 100 μM were subsequently selected as experimental doses of nicotine.

### 3.3. Inflammatory Cytokines and Chemokines in HMC3 Cells

We clarified the changes in inflammatory factor release induced by LPS in HMC3 cells using the Bio‐Plex Suspension Array System to simultaneously detect 27 human‐derived inflammatory factors and chemokines. We screened the relevant inflammatory factors in HMC3 cells impacted by nicotine treatment. The results of the 27 cytokines are shown in Table 2.

**Table 2:** Levels of 27 human‐derived inflammation‐associated cytokines in the supernatant of HMC3 cells.

Indicators	Con	Mod	Low	Med	High	Pc	Nc
Eotaxin	16.88 ± 0.54	18.75 ± 0.83	19.00 ± 1.58	19.50 ± 2.06	18.00 ± 0.71	18.50 ± 1.50	19.50 ± 0.50
Basic FGF	13.25 ± 1.09	14.50 ± 0.50	15.88 ± 1.43	14.75 ± 1.48	14.50 ± 0.50	17.25 ± 2.28	13.75 ± 1.09
G‐CSF	15.50 ± 0.50	16.88 ± 0.22 ^∗^	16.88 ± 0.89	17.50 ± 1.50	16.63 ± 0.65	16.25 ± 0.83	16.00 ± 1.22
GM‐CSF	16.00 ± 0.71	20.25 ± 0.56 ^∗∗∗^	19.75 ± 1.48	19.75 ± 1.09	19.50 ± 0.50	17.63 ± 0.82	20.25 ± 0.83
IFN‐γ	13.75 ± 0.43	14.50 ± 0.50	14.13 ± 1.14	14.25 ± 0.83	14.50 ± 1.12	14.25 ± 1.09	14.50 ± 0.50
IL‐1β	10.75 ± 0.43	12.00 ± 0.71	12.00 ± 0.71	11.25 ± 0.83	11.38 ± 0.65	11.13 ± 0.54	11.50 ± 0.50
IL‐1α	6.75 ± 0.43	9.25 ± 3.09	7.75 ± 0.43	6.63 ± 0.41	7.50 ± 0.50	7.00 ± 0.00	6.88 ± 0.54
IL‐2	25.50 ± 1.50	26.00 ± 0.71	25.75 ± 1.48	27.38 ± 0.41	26.63 ± 1.19	25.50 ± 1.66	24.25 ± 2.25
IL‐4	6.00 ± 0.00	8.25 ± 0.43 ^∗∗∗^	7.25 ± 0.43	8.25 ± 0.83	7.88 ± 0.54	7.75 ± 0.43	8.25 ± 0.83
IL‐5	33.50 ± 0.50	38.25 ± 1.09 ^∗∗^	37.38 ± 1.78	38.25 ± 0.83	37.00 ± 1.58	37.38 ± 3.15	37.25 ± 3.70
IL‐6	247.00 ± 17.85	2134.75 ± 91.17 ^∗∗∗∗^	1750.25 ± 59.54	1675.38 ± 167.80	1421.25 ± 848.53	1378.25 ± 181.27	2073.00 ± 415.06
IL‐7	42.38 ± 2.43	44.88 ± 1.24	46.25 ± 1.92	42.88 ± 1.67	45.38 ± 1.85	43.25 ± 2.17	44.00 ± 2.12
IL‐8	74.63 ± 11.30	824.50 ± 20.57 ^∗∗∗∗^	772.13 ± 76.73	806.38 ± 57.79	827.38 ± 99.26	621.25 ± 61.29	912.00 ± 50.85
IL‐9	26.00 ± 0.00	35.63 ± 1.56 ^∗∗∗∗^	34.50 ± 0.87	33.50 ± 2.29	34.00 ± 0.71	32.00 ± 0.71	33.50 ± 1.50
IL‐10	14.00 ± 0.71	13.88 ± 0.74	14.00 ± 0.00	13.75 ± 0.43	14.13 ± 0.54	13.00 ± 0.00	13.50 ± 1.12
IL‐12(p70)	17.25 ± 0.83	17.50 ± 0.50	17.50 ± 1.12	17.00 ± 1.22	18.00 ± 0.71	17.50 ± 0.50	17.00 ± 1.22
IL‐13	8.88 ± 0.54	9.75 ± 0.43	9.75 ± 0.25	9.00 ± 0.71	9.75 ± 0.43	8.50 ± 0.87	10.00 ± 0.71
IL‐15	37.63 ± 1.85	35.75 ± 1.64	36.25 ± 1.09	36.75 ± 3.11	36.50 ± 2.96	35.50 ± 2.29	35.75 ± 9.28
IL‐17	14.00 ± 0.41	19.25 ± 4.43 ^∗∗^	14.25 ± 0.50	14.25 ± 0.50	14.00 ± 0.82	13.88 ± 0.88	16.25 ± 2.63
IP‐10	13.25 ± 1.48	149.38 ± 25.72 ^∗∗∗∗^	151.00 ± 11.25	138.63 ± 14.52	150.75 ± 27.76	92.38 ± 4.68	169.00 ± 41.79
MCP‐1	34.50 ± 2.06	112.25 ± 7.19 ^∗∗∗∗^	90.25 ± 7.70	98.38 ± 8.17	106.38 ± 3.76	77.75 ± 1.75	107.63 ± 5.96
MIP‐1α	8.38 ± 0.41	8.75 ± 0.83	9.25 ± 0.43	9.25 ± 0.43	9.00 ± 0.71	8.75 ± 0.43	9.00 ± 0.00
MIP‐1β	25.50 ± 0.50	35.88 ± 1.43 ^∗∗^	37.25 ± 1.09	36.75 ± 2.95	37.13 ± 2.01	33.75 ± 1.30	36.75 ± 2.49
PDGF‐BB	29.00 ± 0.71	28.50 ± 0.50	28.75 ± 1.79	27.38 ± 0.96	28.00 ± 1.87	26.25 ± 3.63	28.50 ± 1.54
RANTES	94.75 ± 8.79	149.50 ± 4.30 ^∗∗∗∗^	120.67 ± 5.54	118.50 ± 7.97	123.83 ± 8.27	113.75 ± 7.15	119.75 ± 13.37
TNF‐α	12.75 ± 0.43	14.00 ± 0.71	13.50 ± 1.50	14.63 ± 0.65	13.00 ± 0.71	13.63 ± 0.41	14.50 ± 0.50
VEGF	53.50 ± 4.99	55.13 ± 1.60	49.88 ± 2.46	41.63 ± 2.53	42.88 ± 6.19	45.50 ± 4.15	50.00 ± 11.85

*Note:* Results were presented as mean ± SD. 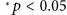, 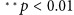, 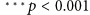, 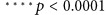 showed significant differences in the model control group compared to the blank control group. Eotaxin: eosinophil chemotactic protein.

Abbreviations: FGF, fibroblast growth factor; G‐CSF, granulocyte colony‐stimulating factor; GM‐CSF, granulocyte‐macrophage colony‐stimulating factor; IFN‐γ, interferon gamma; IL‐1β, interleukin‐1 beta; IL‐1α, interleukin‐1 alpha; IL‐2, interleukin‐2; IL‐4, interleukin‐4; IL‐5, interleukin‐5; IL‐6, interleukin‐6; IL‐7, interleukin‐7; IL‐8, interleukin‐8; IL‐9, interleukin‐9; IL‐10, interleukin‐10; IL‐12(p70), interleukin‐12 p70; IL‐13, interleukin‐13; IL‐15, interleukin‐15; IL‐17, interleukin‐17; IP‐10, interferon gamma‐induced protein‐10; MCP‐1, monocyte chemoattractant protein‐1; MIP‐1α, macrophage inflammatory protein‐1 alpha; MIP‐1β, macrophage inflammatory protein‐1 beta.

ANOVA analysis of the 27 cytokine expression profiles in the supernatant from each group. Results indicated that 13 cytokines, including IFN‐γ, IL‐1β, IL‐1α, IL‐2, IL‐7, IL‐10, IL‐12(p70), IL‐13, IL‐15, TNF‐α, Eotaxin, MIP‐1α, and PDGF‐BB, exhibited significant differences between two groups (*p* < 0.05). However, levels for the remaining 14 cytokines, IL‐4, IL‐5, IL‐6, IL‐8, IL‐9, IL‐17, MCP‐1 (MCAF), RANTES, VEGF, FGF basic, G‐CSF, GM‐CSF, IP‐10, and MIP‐1β, were not significantly different between the groups (*p* > 0.05).

Among these cytokines, LPS induction resulted in a significant increase in 12 cytokines in HMC3 cells compared to the blank control group, including IL‐4, IL‐5, IL‐6, IL‐8, IL‐9, IL‐17, MCP‐1 (MCAF), RANTES, G‐CSF, GM‐CSF, IP‐10, and MIP‐1β (*p* < 0.05).

Two cytokines and three chemokines exhibited modest but significant differences in HMC3 cells after nicotine treatment compared to the model control group, namely IL‐6, IL‐17, MCP‐1 (MCAF), RANTES, and VEGF.

Compared to the model control, both nicotine and GTS‐21 pretreatment inhibited IL‐6 and IL‐17 release, with both 1 and 10 µM nicotine pretreatment causing a significantly lower IL‐6 level than in the model control (*p* < 0.05). While 100 µM nicotine pretreatment resulted in significantly lower IL‐17 levels than the model control (*p* < 0.05) (Figure 3a,b).

Figure 3HMC3 physiological indicators related to inflammatory response. (a) The amount of IL‐6 released into the supernatant of each group of cells. p(Mod vs. Con) <0.0001, p(Mod vs. Low) = 0.0031, p(Mod vs. Med) = 0.0221, p(Mod vs. Pc) = 0.0043. (b) The amount of IL‐17 released into the supernatant of each group of cells. p(Mod vs. Con) = 0.0071, p(Mod vs. Low) = 0.0106, p(Mod vs. Med) = 0.0106, p(Mod vs. High) = 0.0071, p(Mod vs. Pc) = 0.0058. (c) The amount of MCP‐1(MCAF) released into the supernatant of each group of cells. p(Mod vs. Con) <0.0001, p(Mod vs. Low) = 0.0007, p(Mod vs. Med) = 0.0368, p(Mod vs. Pc) <0.0001. (d) The amount of RANTES released into the supernatant of each group of cells. p(Mod vs. Con) <0.0001, p(Mod vs. Low) = 0.0051, p(Mod vs. Med) = 0.0027, p(Mod vs. High) = 0.0125, p(Mod vs. Pc) = 0.0004. (e) The amount of VEGF released into the supernatant of each group of cells. p(Mod vs. Med) = 0.0030, p(Mod vs. High) = 0.0072, p(Mod vs. Pc) = 0.0410. (f) The amount of nitrite formed through spontaneous oxidation of NO in the cell supernatant of each group. (g) Detection of intracellular nitric oxide synthase enzymatic activity in cells treated with LPS for 2, 4, 8, and 24 h. (h) BDNF expression in the supernatant of each group of cells. p(Mod vs. Low) = 0.0107, p(Mod vs. Med) = 0.0002, p(Mod vs. High) <0.0001, p(Mod vs. Pc) = 0.0010. (i) GDNF expression in the supernatant of each group of cells. p(Mod vs. Con) <0.0001, p(Mod vs. Med) = 0.0407, p(Mod vs. High) < 0.0001, p(Mod vs. Pc) = 0.0156.

**Figure:**
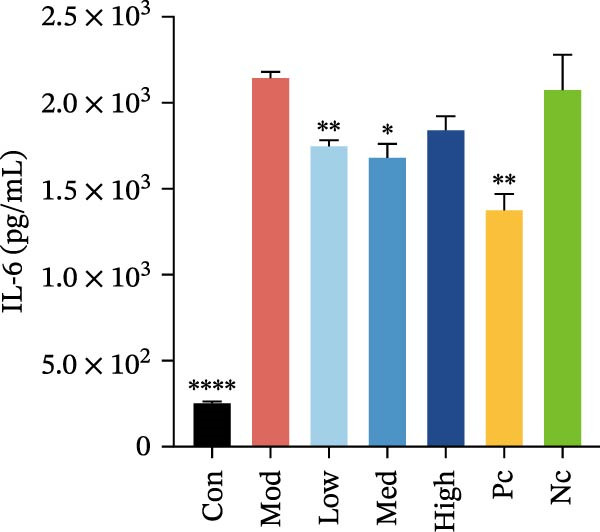
(a)

**Figure:**
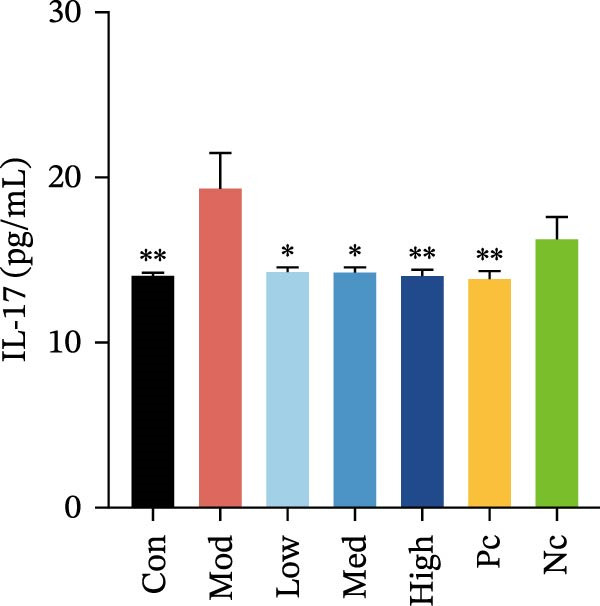
(b)

**Figure:**
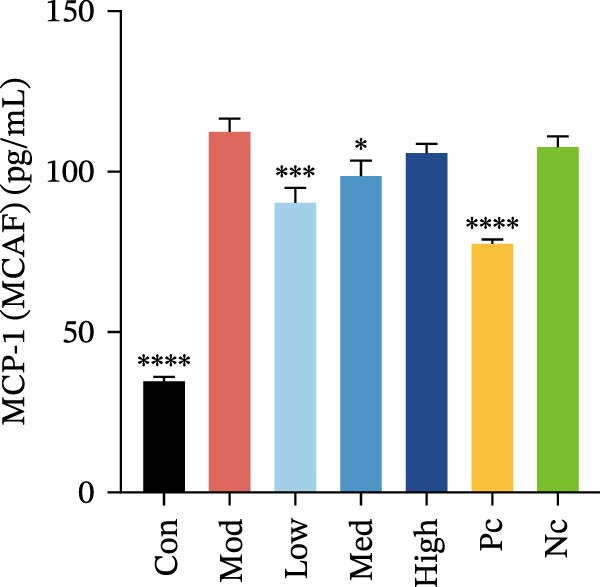
(c)

**Figure:**
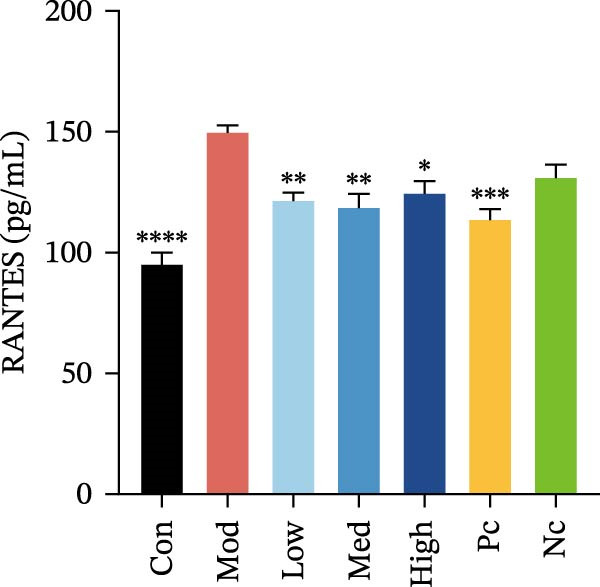
(d)

**Figure:**
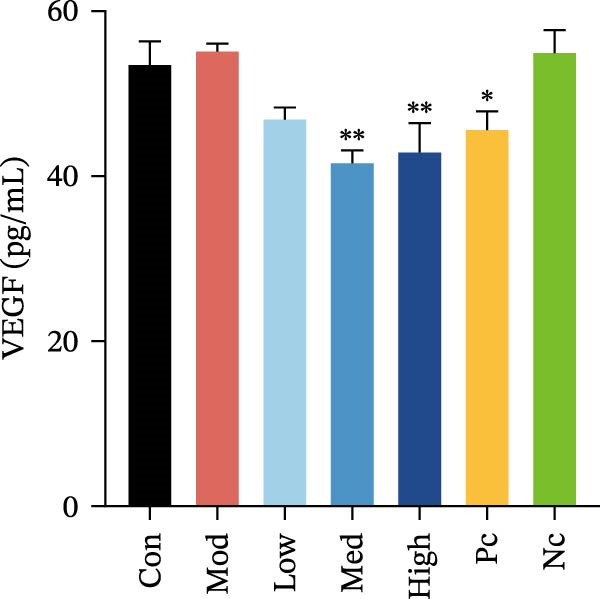
(e)

**Figure:**
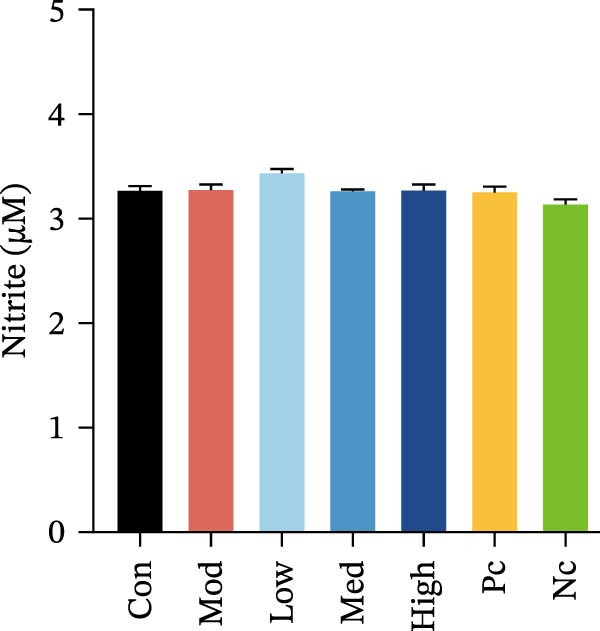
(f)

**Figure:**
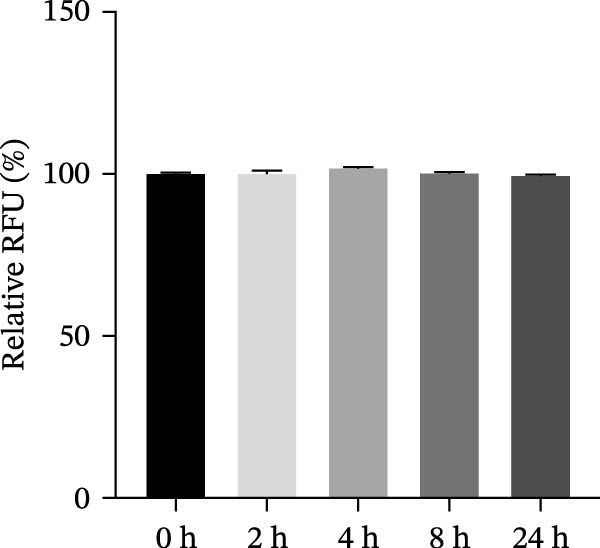
(g)

**Figure:**
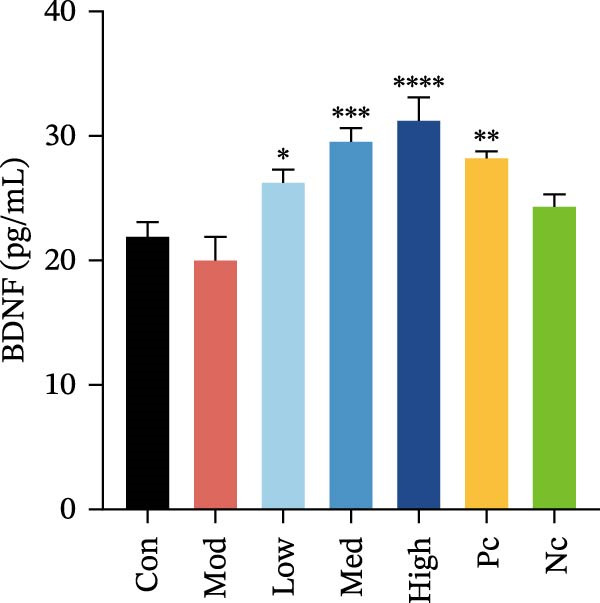
(h)

**Figure:**
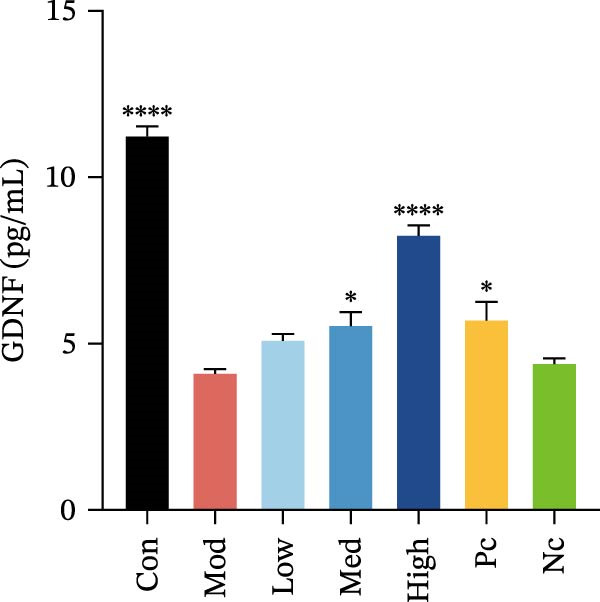
(i)

Compared to the model control group, both nicotine and GTS‐21 pretreatment reduced levels of MCP‐1 (MCAF), RANTES, and VEGF release. MCP‐1 (MCAF) levels after 1 μM nicotine and 10 μM nicotine pretreatment were significantly lower than the model control group (*p* < 0.05) (Figure 3c). RANTES levels were significantly lower after exposure to 1 μM nicotine, 10 μM nicotine, and 100 μM nicotine than in the model control group (*p* < 0.05) (Figure 3d). VEGF levels were significantly lower after treatment with 10 μM nicotine than in the model control group (*p* < 0.05) (Figure 3e).

Based on these results, we observed a significant decrease in these two cytokines and three chemokines after exposure to GTS‐21. This indicates that activation of α7 nAChR may induce changes in these inflammatory factors in HMC3 cells. After nicotine pretreatment, the findings are consistent with GTS‐21, indicating that nicotine also interacted with α7 nAChR, a key target for inflammatory inhibition in HMC3 cells.

### 3.4. NO Release and NOS Activity in HMC3 Cells

To determine whether LPS induction enhances NOS enzymatic activity and subsequent NO release in HMC3 cells, we collected cell supernatants to detect nitrite, a stable oxidative product of NO, in the supernatants was measured using the Griess method, while NOS activity in the cells was evaluated using a fluorescent probe method.

After 24 h of treatment according to the experimental groups, supernatant nitrite levels were quantified. The results showed no significant increase in nitrite in the LPS model group compared to the blank control group (*p* > 0.05), suggesting that LPS induction did not elevate NO levels in HMC3 cells (Figure 3f). To rule out the possibility that the lack of nitrite detection was due to the duration of LPS exposure, we further conducted a time‐course experiment with induction periods of 2, 4, 8, and 24 h to examine potential temporal changes in NO synthase activity. The results demonstrated that the NO synthase activity did not increase alongside the time of LPS induction in HMC3 cells (*p* > 0.05) (Figure 3g). Together, these findings indicate that under our experimental conditions, LPS did not stimulate NO production in HMC3 cells, and nicotine treatment did not alter the NO expression profile.

### 3.5. Neurotrophic Factor Expression in HMC3 Cells

To determine whether nicotine exposure results in the production of neurotrophic factors in HMC3 cells, we collected cellular supernatants to detect the expression of BDNF and GDNF using an enzyme‐linked immunosorbent assay. The results showed that LPS induction led to a significant decrease in GDNF levels in HMC3 cells compared to the blank control group. In contrast, nicotine intervention increased GDNF expression relative to the LPS model group (*p* < 0.05). This enhancement was abolished when the α7 inhibitor MLA was applied, reducing GDNF levels to those observed in the model control group (Figure 3i). In the case of BDNF, LPS induction did not significantly alter its levels compared to the model control group in HMC3 cells (*p* > 0.05). However, both the nicotine‐treated group and the positive control group exhibited a marked increase in BDNF expression (*p* < 0.05). Similarly, the addition of MLA counteracted this effect, bringing BDNF levels back to those of the model control group (Figure 3h).

Both nicotine and the selective α7 nAChR agonist elevated the expression of BDNF and GDNF expression in HMC3 cells. This suggests that α7 nAChR activation may promote neurotrophic factor expression in HMC3 cells, which contributes to enhanced support for peripheral neuronal function.

### 3.6. The mRNA Expression of α7 nAChR in HMC3 Cells

HMC3 cells were pretreated with nicotine and the α7 nAChR‐specific agonist GTS‐21 for 1 h, followed by LPS induction for 2 h. Total intracellular RNA was then extracted and analyzed via qPCR. The results showed that LPS stimulation did not significantly increase the transcript level of α7 nAChR compared to the blank control group (*p* > 0.05). In contrast, treatment with nicotine at concentrations of 1, 10, and 100 μM significantly upregulated the mRNA expression of α7 nAChR (*p* < 0.05), with effects comparable to those observed with 100 μM GTS‐21. Notably, the upregulation did not follow a dose‐dependent pattern, suggesting that even the lowest concentration of nicotine (1 μM) was sufficient to activate the majority of α7 nAChRs in HMC3 cells. Conversely, after treatment with the α7 nAChR‐specific antagonist, MLA, the mRNA expression of α7 nAChR was significantly suppressed, approaching baseline levels (Figure 2a). These results demonstrate that nicotine intervention significantly enhances the transcriptional expression of α7 nAChR mRNA in HMC3 cells.

### 3.7. The Protein Expression of PI3K in HMC3 Cells

According to our qPCR results, nicotine at concentrations of 1, 10, and 100 μM induced comparable activation of α7 nAChR mRNA expression. Therefore, a concentration of 10 μM nicotine was selected as the experimental concentration in WB experiments. After 4 h of administration according to our experimental design, protein extracts were harvested to assess the changes in the PI3K phosphorylation level (Figure 2b). Densitometric analysis of the immunoblot bands revealed that the p‐PI3K/PI3K level in the LPS model group showed a slight increase compared to the blank control group, although this difference was not statistically significant (*p* > 0.05). In contrast, the nicotine treatment group exhibited a significant increase in p‐PI3K/PI3K compared to the LPS model group (*p* < 0.05). This enhancement was abolished by the addition of the α7 nAChR inhibitor, MLA, which reduced the p‐PI3K/PI3K to a level comparable to that of the LPS model group (*p* > 0.05) (Figure 2c).

These results suggest that nicotine may promote PI3K phosphorylation through activation of α7 nAChR, an effect that is reversible upon inhibition with MLA, indicating that activation of α7 nAChR impacts PI3K protein. The modest increase in PI3K phosphorylation observed in the LPS‐treated group may reflect a compensatory cellular response to inflammatory stress.

Furthermore, our findings underscore the importance of optimizing the time point for protein detection in signaling studies. Previous experiments found that the phosphorylation level of PI3K was detectable as early as 2 h after treatment, but reached higher levels at 4 h. No change in the phosphorylation level of this protein was observed at later time points (8–24 h). This highlights that the kinetics of regulatory protein expression are cell type‐ and stimulus‐dependent, and should be empirically determined through time‐course experiments.

### 3.8. The Neuronal Activity of SH‐SY5Y in a Coculture System

To simulate neuroinflammation in the central nervous system, a transwell coculture system was established using HMC3 microglial cells and SH‐SY5Y neuronal cells. In the nicotine intervention group, HMC3 cells were pretreated with nicotine for 1 h. Subsequently, HMC3 cells were stimulated with LPS for 24 h. Neuronal activity of SH‐SY5Y neurons was then measured using the MTT assay. The results demonstrated that the neurons viability in the LPS‐treated group was significantly inhibited to approximately 74.3% compared to the blank control group (*p* < 0.05). In contrast, nicotine pretreatment markedly restored neuronal activity to approximately 85.7% relative to the LPS model group (*p* < 0.05) (Figure 2d). These results suggest that nicotine pretreatment attenuates LPS‐induced inflammatory responses in HMC3 cells, and such modulation of the immune microenvironment may contribute to the preservation of neuronal activity.

## 4. Discussion

In this study, the antineuroinflammatory effects of nicotine were systematically investigated through multiple experimental approaches. Coculture assays demonstrated the neuroprotective potential of nicotine and elucidated underlying signaling mechanisms. Specifically, nicotine was found to suppress the release of several inflammation‐related cytokines and chemokines in HMC3 cells and promoted the expression of BDNF and GDNF. In addition, in the microglia‐neuron coculture system, nicotine treatment significantly enhanced neuronal survival under inflammatory conditions. The study found that nicotine probably regulated PI3K phosphorylation through α7 nAChR, which may be a plausible molecular basis for its neuroprotective action.

The human‐derived microglia cell line HMC3 was selected as the experimental model in this study. While numerous studies have employed murine‐derived microglia lines, research on human‐derived microglia remains limited, primarily due to challenges in sourcing primary human microglia and obtaining sufficient quantities [20]. LPS, a component of the outer cell wall of Gram‐negative bacteria, is commonly used to trigger a cascade of immune activation and induce inflammation responses across various cells, tissues, and organs. However, relatively few studies have focused on the inflammatory response in human‐derived microglia, and even fewer have explored the modulatory effects of nicotine on such responses.

The use of HMC3 cells, a human‐based model, offers clinically relevant insights that may facilitate future translational studies of the human nervous system. Moreover, HMC3 represents a candidate for investigating the impact of LPS on activation phenotype and inflammatory level. In addition, we implemented a nicotine intervention to investigate whether it modulates inflammatory response in HMC3 cells through α7 nAChR signaling, thereby helping to elucidate its potential mechanism of action.

Previous studies in microglial biology have largely utilized BV2 cells, focusing on inflammatory markers that reflect the functional state of microglia [21, 22]. Microglia are known to adopt two activation phenotypes: the classically activated pro‐inflammatory phenotype and the alternatively activated anti‐inflammatory phenotype. To better understand the functional shifts in microglial behavior, numerous investigations have therefore aimed to characterize these polarization states across different cellular models [23]. For instance, Qichun Zhang et al. [24] demonstrated that acetylcholine‐mediated activation of α7 nAChR in BV2 cells promoted a transition from the pro‐inflammatory to the anti‐inflammatory phenotype. In the present study, we examined a variety of inflammation‐related cytokines and chemokines, along with NO synthase activity and NO release, to comprehensively assess the microglial inflammation. We also performed some tests using BV2 cells to facilitate comparison.

To profile inflammatory mediators, we employed a suspension microarray assay. This approach enabled us to identify previously unreported roles of nicotine in the regulation of specific inflammatory factors and chemokines. Our findings demonstrated that LPS induced a significant elevation in 12 pro‐inflammatory chemokines, suggesting the sensitivity of HMC3 cells to LPS stimulation. Notably, nicotine treatment led to a modest but statistically significant reduction in 2 cytokines and 3 chemokinesnamely IL‐6, IL‐17, MCP‐1 (MCAF), RANTES, and VEGF, while no upregulation was observed in the other factors. IL‐6 and IL‐17 are well‐characterized pro‐inflammatory factors [25, 26]. Monocyte chemotactic protein‐1 activates monocytes and microglia, promoting the secretion of inflammatory mediators. RANTES regulates T‐cell‐mediated immune responses, facilitates leukocyte migration and infiltration, and may modulate cell growth and differentiation. VEGF interacts with cytokines and promotes inflammatory responses [27, 28]. Overall, nicotine reduced the expression of MCP‐1, RANTES, and VEGF in HMC3 cells. These factors are implicated in the regulation of microglia, chemotactic protein aggregation, and inflammatory cell infiltration. Their reduced secretion suggests that nicotine may attenuate microglia‐mediated inflammation by limiting the recruitment and activation of immune cells at the site of injury.

In our experiments, LPS induction did not alter NO synthase activity or increase NO release in either HMC3 or BV2 cells. This finding contrasts with several published studies. For example, Marta Garcia‐Contreras’ team detected a significant upregulation of iNOS expression in HMC3 cells [29], Feng Jiang et al. [30] reported increased iNOS and NO release in HMC3 cells. Similarly, Zhao et al. [31] described dynamic time‐dependent changes in iNOS expression in primary microglia in response to LPS, with the highest expression observed after 24 h. However, according to Russo et al. [32] in a published review, nitrite levels in HMC3 cells remained undetectable under basal conditions and after 24‐h exposure to various pro‐inflammatory stimuli, including LPS, LPS/IFN‐γ, IL‐1β, TNF‐α, either alone or in combination with IFN‐γ. Moreover, resting HMC3 cells did not express iNOS, and its expression is not induced by pro‐inflammatory activation, which is consistent with our experimental observations. The HMC3 cell line, a valuable model for studying human microglial inflammation, has been consistently shown in the literature to produce pro‐inflammatory cytokines robustly in response to LPS but to exhibit minimal to no iNOS/NO response under standard stimulation protocols [33], which might lead to the result of NO in our experiment.

As an immune cell, microglia play a pivotal role in regulating the immune microenvironment of the brain. One of their essential functions is the secretion of trophic factors, which regulate neuronal survival. In our study, the expression of BDNF and GDNF in HMC3 cells was examined, and nicotine stimulation promoted BDNF expression and attenuated inflammation in HMC3 cells, which was consistent with previous reports [34, 35]. In addition, our results demonstrated that nicotine increased expression of GDNF, suggesting a possible role in modulating microglial activation through indirect or multicellular mechanisms. The absence of a significant change in BDNF expression following LPS stimulation alone, despite a clear inflammatory response, is a notable observation. This might be caused by the cell type specificity and stimulation specificity regulation of neurotrophic factors. While LPS is a potent inducer of pro‐inflammatory cytokines in microglia, its effect on BDNF is highly variable and context‐dependent. Several studies using human microglial cells or cell lines have reported that LPS does not necessarily suppress BDNF or may even induce it under certain conditions, unlike the more consistent suppression often observed in neuronal cultures or in vivo models, where the net effect reflects a complex cellular interplay [36]. The baseline expression level of BDNF in HMC3 cells might be relatively resilient to the specific inflammatory signals triggered by our LPS treatment paradigm. Conversely, the significant decrease in GDNF aligns with literature showing its greater sensitivity to inflammatory suppression.

In the present study, we focused on the protective role of nicotine against neuroinflammation and demonstrated that its anti‐inflammatory effects are mediated primarily through the α7 nAChR‐PI3K signaling pathway. As a key component of the cholinergic anti‐inflammatory pathway, α7 nAChR is expressed on the surface of immune cells and mitochondria, where it can be activated by ligands such as acetylcholine and nicotine to regulate the influx of Ca^2+^ [37]. It has been demonstrated across numerous studies that it plays an important Extensive evidence supports its critical role in immunity regulation, and α7 nAChR agonists are currently under intense investigation as potential therapeutic agents [38, 39].

The PI3K/AKT signaling pathway plays a critical role in regulating downstream effectors to mitigate neurotoxicity and treat neurodegenerative diseases. It is also involved in regulating immune responses and contributes to inflammatory processes of various diseases [40–42]. Among the four isoforms of PI3K, targeting the PI3Kδ isoform has been shown to suppress the production of pro‐inflammatory cytokines [43]. Natural compounds, such as those derived from burdock seeds, have also been reported to attenuate cytokine and chemokine release by modulating the PI3K/Akt pathway [44]. Accumulating evidence underscores the versatile functions of PI3K in coordinating cellular responses and counteracting detrimental inflammatory responses [45–48]. Furthermore, nicotine has been shown to activate PI3K signaling via activation of α7 nAChR, which has been observed in studies of peripheral inflammatory diseases [6, 49]. Together, these findings suggest that the α7 nAChR/PI3K pathway may represent a promising therapeutic target for the treatment of neuroinflammation and related neurological disorders.

To evaluate the neuroprotective effect of nicotine, we established a coculture system comprising human‐derived microglia and neuronal cells. Coculture models are often used in the study of neurological diseases to better recapitulate disease‐relevant microenvironments. For instance, in Alzheimer’s disease, Luchena et al. [50] developed a triple coculture model consisting of mouse astrocytes, neurons, and microglia. Similarly, Lopez‐Lengowski et al. [51] generated microglia from human induced pluripotent stem cells and cocultured them with cortical neurons to study neuron–microglia interactions. In another study, Beaulieu et al. [52] constructed a murine microglia–neuron coculture system using transwell chambers to investigate anti‐inflammation of oleic acid, wherein N9 microglia communicated with PC12 neurons through paracrine signaling—a methodology analogous to that used in our study. In our study, HMC3 microglia and SH‐SY5Y neurons were cultured in transwell chambers, which allowed us to specifically examine how nicotine‐pretreated microglia indirectly influence neuronal survival. The results demonstrated a significant increase in neuronal viability, supporting the conclusion that nicotine exerts neuroprotective effects through modulation of microglial function.

This study established an in vitro cocellular model of neurons and microglia to mimic neuroinflammation and investigate potential interactions between microglia and neurons. In the future, we will attempt to select different functional neurons for the study of the specific immunomodulatory effects of microglia on neuronal functionality. Moreover, we will continue in vivo animal experiments, employing different neurological disease models to more comprehensively and precisely evaluate the pharmacological effects of nicotine.

## 5. Conclusions

In this study, human microglial HMC3 cells were employed to establish an LPS‐induced inflammation model, which was subsequently used to evaluate the anti‐inflammatory effects of nicotine. The results showed that treatment with a low concentration of nicotine not only attenuated inflammation but also enhanced the secretion of BDNF and GDNF. Consequently, in a microglia neuron coculture system, this modulation of the inflammatory microenvironment resulted in reduced neuronal apoptosis. We propose that nicotine, as an exogenous agonist of α7 nAChR, mediates anti‐inflammatory effects primarily through the nAChR/PI3K signaling pathway. Collectively, our findings reveal the immunomodulatory potential of low concentrations of nicotine within the central nervous system and highlight its indirect neuroprotective properties, suggesting possible therapeutic applications.

## Author Contributions

Yuhan Qin performed, analyzed the experiments, and wrote the original manuscript. Xiaohui Yan performed, analyzed the experiments, and revised the manuscript. Yan‐Bo Luo designed, supervised, and revised the manuscript. Hongjuan Wang, Yushan Tian, and Xiaqing Wu revised the manuscript. Tao Zhang revised the manuscript. Hongli An designed and revised the manuscript. Huan Chen, Hongwei Hou, and Qingyuan Hu designed and revised the manuscript and provided funding support.

## Funding

This work was supported by the Provincial and Ministerial Major Project of China (Grants 110202102011 and 110202101018 (XX‐04)), Henan Province Science and Technology Research Project (Grant 242102310399), and Beijing Life Science Academic (Grants 2023000CA0050 and 2023000CC0160).

## Disclosure

All authors have read and approved the final version of the manuscript. An earlier version of this article appeared as a preprint on the Research Square platform [53].

## Consent

The authors have nothing to report.

## Conflicts of Interest

The authors declare no conflicts of interest.

## Data Availability

The data related to this article may be shared by the corresponding author upon reasonable request.
